# Chitosan-based liposomal thermogels for the controlled delivery of pingyangmycin: design, optimization and *in vitro* and *in vivo* studies

**DOI:** 10.1080/10717544.2018.1444684

**Published:** 2018-02-27

**Authors:** Ling Zhang, Fen Chen, Jiatong Zheng, Hongwei Wang, Xingjun Qin, Weisan Pan

**Affiliations:** a Department of Biotherapy, Cancer Research Institute, The First Affiliated Hospital of China Medical University, Shenyang, China;; b Key Laboratory of Ministry of Education for TCM Viscera-State Theory and Applications, Liaoning University of Traditional Chinese Medicine, Shenyang, China;; c Department of Pharmaceutics, School of Pharmacy, Shenyang Pharmaceutical University, Shenyang, China;; d Department of Oromaxillofacial Head and Neck Oncology, Ninth People’s Hospital, College of Stomatology, Shanghai Jiao Tong University School of Medicine, Shanghai Key Laboratory of Stomatology, Shanghai, China

**Keywords:** Pingyangmycin, liposomal thermogels, chitosan, optimization, Box-Behnken

## Abstract

Pingyangmycin (PYM) has been applied clinically for many years to treat vascular malformations (VM) in China. The major limitation of PYM injections is quick diffusion from the injection site, which increases side effects, especially the possibility of pulmonary injury. In this paper, chitosan/glycerophosphate disodium (CS/GP) thermogels containing liposomes for sustained and localized PYM delivery were prepared and optimized by a three-level three-factorial Box–Behnken experimental design to evaluate the effects of different variables (the PYM concentration, CS amount and GP content), on the selected responses (cumulative percentage PYM released in 1 day, 9 days and the rate constant *k*). The results revealed that the optimized PYM liposomal thermogels had a controlled PYM release for 14 days *in vitro*, which confirmed the validity of optimization. *In vitro* morphological observation, cell cycle and apoptosis analysis showed an effective anti-proliferation action of PYM liposomal thermogels on human vascular endothelial cells (EA.hy926). *In vivo* pharmacokinetics research in rabbits displayed that compared with PYM liposomes and PYM thermogels, PYM liposomal thermogels had a better controlled delivery of PYM. Histological examination of rabbit ear veins showed that after local application with PYM lipsomal thermogels for 21 days, obvious vein thrombosis and inflammatory reaction could be observed. The above results indicated that PYM-loaded lipsomal CS/GP thermogels might have a good prospect for the treatment of VM.

## Introduction

Hydrogel is composed of network polymers dispersed in a lot of water, which has been widely concerned in the field of biomedical research for its good formulation stability and bio-compatibility (Dou et al., 2014; Zheng et al., 2015). Currently, the preparation of *in situ* gel through a simple sol-gel method with no chemical reaction is a focus in the research for hydrogel, which allows the hydrogel more suitable for the application in controlled drug delivery and biomedical engineering (Wu et al., 2016; Yu et al., 2017). There are many advantages of the biodegradable injectable *in situ* gel system, such as *in situ* drug depot can be formed without surgery treatment and the drug-loading or dose adjustment is convenient (Jiang et al., 2016; Wu et al., 2016). Chitosan (CS) has been widely used in the field of pharmaceutics, medicine and tissue-engineering, because of its bio-degradability, low toxicity and good bio-compatibility. An interesting sol-gel phase transition of the combination of CS and glycerol phosphate disodium (GP) has been reported (Chenite et al., 2000; Supper et al., 2014). The injectable CS/GP thermogels were used as biomaterials for angiogenesis, fiber-cartilage regeneration and bone tissue repair (Cheng et al., 2017). Paclitaxel-loaded CS-based thermogels were studied by intra-tumor injection into tumor-bearing mice, whose findings displayed that compared with Taxol injections, the paclitaxel-loaded thermogels could more effectively inhibit tumor growth and reduce systemic toxicity (Mahajan et al., 2016).

Pingyangmycin (PYM), isolated from streptomycete in the soil of Pingyang county, is a new type of hydrophilic glycopeptide anti-tumor antibiotic. PYM has been applied in clinical in the Far East for over 30 years for treatment of Vascular malformation (VM) and different types of cancer of epidermal origin with an exact therapeutic effect (Chen et al., 2010; Ochiai et al., 2016). VM, which often occurs in oral and maxillofacial region, is a common disease and can be cured by many methods including embolization therapy, sclero-therapy, laser cosmetic treatments, cryo-therapy and radiation therapy (Zhang et al., 2013; Zhao et al., 2013; Li et al., 2017). Best treatment strategies are related to the size and location of the lesion, for example, laser photocoagulation therapy would be adopted for the respiratory disease, while surgical treatment is the best choice for resectable lesion (Urban et al., 2014). Over the years, the treatment of VM mainly focused on the traditional plastic surgery with its shortcomings for instance, visible scar, bad therapeutic dependence, and high risk resulted from the complexity of blood vessels distributed in oromaxillofacial region (Mohan et al., 2015). In addition, the potential facial distortion brought by a surgery may result in mental problems for patients (Zhao et al., 2012). Local and intravenous injection of PYM can lead to endothelial cell injury, thicken the blood vessel wall and vascular occlusion (Luo & Gan, 2013). Now, PYM lyophilized powder for injection is the only commercial preparation. While, short half-life and lots of side effects (e.g. lung toxicity) of PYM have limited its wide application (Yang et al., 2012). As a substitute for traditional surgery, interventional embolization treatment for VM has been utilized more accurately and adaptably to block blood vessels in the past few years (Abdel Aal et al., 2017). Chemo-embolization method, which combines the advantages of embolic materials and therapeutic drugs, has become one of the promising treatments for VM recently (Ashrafi et al., 2017). So, local and slow-release PYM formulation research is greatly essential to improve the therapeutic effect and reduce the adverse effects at the same time. There are some reports on the combination of PYM and bio-degradable embolic materials, such as PYM-loaded PLGA microspheres, PYM-loaded Zein/Zein-sucrose acetate isobutyrate thermogels and PYM-loaded Bovine serum albumin microspheres (Gao et al., 2007; Wang et al., 2008; Han et al., 2010).

In our previous research, an injectable liposome was successfully obtained and optimized for the sustained release of PYM (Zheng et al., 2015; Chen et al. 2017). However, the preparation of PYM-loaded liposomes was critically challenging due to the limited local delivery of PYM in injection site, which could increase its side effects, especially the possibility of pulmonary injury. The incorporation of PYM liposomes with *in situ* thermogels is an effective strategy to overcome the above problem. Sustained and localized PYM delivery can be significantly improved by dissolving it in the liposomal thermogels system.

In the present study, thermosensitive *in situ* gel composed of CS and GP was investigated for PYM-loaded liposomes delivery in chemoembolization for VM, which could switch from solution into a semisolid embolic agent occluding blood vessels as temperature raise, in order to block nutrition supplement and release PYM-loaded liposomes sustainedly to maintain an effective therapeutic concentration. Herein, PYM-loaded liposomal CS/GP-based *in situ* gels were prepared and evaluated *in vitro* and *in vivo*. The effects of considered variables were investigated by the application of a three-level three-factorial Box–Behnken experimental design and the formulation variables were optimized by the utilization of response surface methodology (Imam et al., 2017). This study laid the foundation for developing a PYM delivery system connecting the *in situ* thermogelling peculiarity of CS/GP with the advantages provided by liposomes.

## Materials and methods

### Materials

Pingyangmycin (PYM) was purchased from Di Kang pharmaceutical Co., Ltd. (Liaoyuan, China). Soybean phospholipids and cholesterol were obtained from Shanghai Aikang Co, Ltd (Shanghai, China). Chitosan (CS, 50 kDa) was obtained from Haidebei Marine Bioengineering Co., Ltd. (Jinan, China). Glycerophosphate disodium (GP) was acquired from Sinopharm Chemical Reagent Co., Ltd. (Shenyang, China). Purified water was used after deionization and filtration in a Millipore® system. The human vascular endothelial cell line EA.hy926 was provided by The First Affiliated Hospital of China Medical University (Shenyang, China). Dulbecco’s modified Eagle’s medium (DMEM) was purchased from GIBCO Co., Ltd. (Shanghai, China). Fetal bovine serum (FBS) was the product of Beyotime Biotechnology (Shenyang, China). All other chemical reagents and solvents used were of analytical grade.

### Preparation of PYM liposomes, PYM thermogels and PYM liposomal thermogels

In the present study, PYM-loaded liposomes were produced using the ammonium sulfate gradients technique (Zheng et al., 2015; Chen et al., 2017). Firstly, soybean phospholipids (100 mg) and cholesterol (22.2 mg) were added into flask with 10 ml ethyl alcohol solution. A layer of uniform phospholipid membrane was formed by the rotary-film evaporation method in a 42.3 °C water bath. After vacuum dry for 24 hours, 250 mmol/L ammonium sulfate solution (10 ml) was added to elute the lipid membrane, and then following the probe ultrasonic processing. The suspension of lipids was extruded through a 0.22-µm microporous membrane and then following incubation with PYM (18.3 mg) solution about 40 min in a 42.3 °C water bath. PYM-loaded liposomes thus obtained were stored at room temperature for further research. The starting conditions of PYM liposomes before embedding in thermogels were tested in our previous study and listed in Table 1 (Zheng et al., 2015; Chen et al., 2017).

**Table 1.** The starting conditions of PYM liposomes before embedding in thermogels.

spc:chol (w/w)	PYM:lipid (w/w)	Liposome size (nm)	Encapsulation efficiency (%)	Zeta potential (mV)
4.5	0.15	136 ± 18	63.7 ± 1.59	−28.55 ± 6.81

The ‘cold’ method was adopted for preparation of PYM thermogels and PYM liposomal thermogels. *In vitro* evaluation of gelation time and viscosity of CS/GP thermogels in our previous research showed that the better starting values for CS and GP were 1.8%(w/v) and 6%(w/v), respectively (Chen et al., 2014). Typically, CS (1.8%–2.2%, w/v) powder was mixed with 0.1 mol/L acetic acid with agitation, and then, the mixture was kept stirring overnight to clarified. Then, the CS solution was cooled to 4 °C. GP (6%–14%, w/v) solution was prepared and kept at 4 °C for a period of 10 min. Then, GP solution was dropwise added into CS solution with agitating, and the mixture was stirred for 10 min. Finally, the PYM thermogels or PYM liposomal thermogels was acquired by mixing PYM solution or PYM liposomes with CS/GP solution with agitating for 10 min.

### *In vitro* release studies of PYM formulations

Firstly, 5 mL of the PYM liposomes (placed in a preswollen dialysis bag with a 12,000–14,000 Damolecular weight cutoff), PYM thermogels or PYM liposomal thermogels was placed in 10 mL glass vials (internal diameter about 20 mm) and warmed up in 37 °C water bath for 30 min with the aim of complete transition to gel. Then, 3 mL of PBS (pH 7.4) containing NaN_3_ (0.05%, w/v) was added into the above vials. The vials were placed at 37 °C with the shaking rate of 50 rpm. At predetermined time, the dissolution medium was withdrawn for analysis and replenished with equal volume of fresh medium. The drug concentration in the medium was determined as previously reported (Chen et al., 2014). Each dissolution test *in vitro* was operated in triplicate, then the accumulative release ratio of drug from PYM liposomes, PYM thermogels or PYM liposomal thermogels was calculated.

### Box–Behnken experimental design

A three-level three-factorial Box–Behnken experimental design (Design-Expert, Version 7.1.3, USA) was carried out to assess the effects of the chosen factors including the loading of PYM, CS amount and GP content on the responses of the PYM release behavior *in vitro*, to optimize the preparation parameters of PYM-loaded liposomal CS/GP thermogels. This design applied to the exploration in quadratic response surfaces and the construction of second order polynomial models. The quadratic function produced by the experimental design was of the equation as follow:

$${\text{Y =β}}_{\text{0}}{\text{+β}}_{\text{1}}{\text{X}}_{\text{1}}{\text{+β}}_{\text{2}}{\text{X}}_{\text{2}}{\text{+β}}_{\text{3}}{\text{X}}_{\text{3}}{\text{+β}}_{\text{12}}{\text{X}}_{\text{1}}{\text{X}}_{\text{2}}{\text{+β}}_{\text{23}}{\text{X}}_{\text{2}}{\text{X}}_{\text{3}}{\text{+β}}_{\text{13}}{\text{X}}_{\text{1}}{\text{X}}_{\text{3}}{\text{+β}}_{\text{11}}{\text{X}}_{\text{1}}{}^{\text{2}}{\text{+β}}_{\text{22}}{\text{X}}_{\text{2}}{}^{\text{2}}{\text{+β}}_{\text{33}}{\text{X}}_{\text{3}}{}^{\text{2}}$$

where Y is the measured response associated with the combination of every factor level, β_0_ is the intercept, β_i_’s (for i = 1–3) are the linear effects, β_ii_’s (for i = 1–3) the quadratic effects, β_ij_’s (for i, j = 1–3, i < j) the interaction between the ith and jth variables (Irani et al., 2017). The factors selected and settings of factor levels were listed in Table 2.

**Table 2.** Factors and responses in Box–Behnken design.

Factors	Levels		
	−1	0	+1
X_1_ PYM (mg/mL)	2	4	6
X_2_ CS % (w/v)	1.8	2.0	2.2
X_3_ GP % (w/v)	6	10	14
Responses		Constraints	

Y_1_% Release of PYM in 1 day		Minimize	
Y_2_% Release of PYM in 9 days		Maximize	
Y_3_ Rate constant k		Maximize	

X_1_ is the factor of durg loading of the peraration, X_2_ is the factor of CS amount, X_3_ is the factor of GP content. Y_1_ is the response of cumulative release percentage of PYM in 1 day, Y_2_ is the response of cumulative release percentage of PYM in 9 days, Y_3_ is the response of rate constant.

### *In vitro* cytological evaluation

#### Cell culture

EA.hy926 cells were cultured in DMEM Medium supplemented with 10% fetal bovine serum at 37 °C in a humidified 5% CO_2_ incubator. The cells were used for analysis at 3–7 passages.

#### Cell morphology study

An inverted fluorescence microscope was used to observe the morphology change of EA.hy926 cells after incubation with blank liposomal thermogels, PYM liposomes and PYM liposomal thermogels (equaled to 10 μg/mL PYM). EA.hy926 cells were cultured in 24-well plates at a cell density of 2 × 10^4^ cells per well. A matched group was also utilized through the whole experiment. After post treatment process, cells were stained by hematoxylin and eosin (H&E), and fluorescent Hoechst 33258, respectively. Each well was tested with the microscope at 200 × and 400 × magnification, and each group got three micrographs by using a video capture system.

#### Cell cycle analysis and apoptosis assay

EA.hy926 cells were seeded in 24-well plates (1 × 10^4^ cells/well) and cultured with DMEM for 24 h. Medium was then replaced by 3 mL of two different concentrations of PYM liposomes or PYM liposomal thermogels, and continued to incubate for another 24 h & 48 h to reach 80% confluency. Then the cells were harvested and washed twice with ice-cold PBS. All cells were firstly divided to two parts for different assays. One part was analyzed for cycle distribution, and the steps were summarized as follows: cells were fixed and permeabilized with 70% cold ethanol, then stained with PI and analyzed by using a flow cytometer (BD Biosciences, San Jose, CA). The percentage of cells in different cell cycle phases (G0/G1, S and G2/M phase) was calculated using CELL Quest software. The other part was analyzed for apoptosis and necrosis, details were as follows: cells were harvested and washed with ice-cold PBS, stained with Annexin V-FITC and PI according to the manufacturer’s instructions of Annexin V-FITC/PI KIT. Finally, the samples were also analyzed by flow cytometer. Cells in media without PYM (negative control) and cells treated with blank liposomal thermogels were also analyzed as well.

### *In vivo* pharmacokinetic study in rabbits

#### Design of pharmacokinetic experiments

New Zealand rabbits were randomly divided into three groups (*n* = 6) and received an injection via auricular brim veins of PYM liposomes, PYM thermogels or PYM liposomal thermogels (PYM 10 mg/kg). Blood samples were collected from another ear marginal vein of rabbits to heparinized tubes at predetermined time intervals obtaining the plasma after centrifugation (8 min, 10000 rpm). The plasma was stored at −20 °C for further analysis.

#### Plasma sample analysis

The plasma sample processing method was used in our previous research and analyzed using a HPLC method (Chen et al., 2014). Briefly, after naturally melting at room temperature, the plasma sample was mixed with caffeine and trichloroacetic acid solution, and then centrifuged at 10,000 rpm for 10 min. The collected supernatant was injected into sampling valve for HPLC analysis. Pharmacokinetic parameters such as the area under the curve (AUC) and half-life were calculated by using DAS 2.1.1 Pharmacokinetic Software Version.

### *In vivo* chemoembolization studies in rabbits

Twenty New Zealand rabbits were used for *in vivo* chemoembolization studies, which were divided randomly into four groups (*n* = 5), including the groups of normal saline, blank liposomal thermogels, PYM liposomes and PYM liposomal thermogels. The above formulations were slowly injected via auricular brim veins (PYM 10 mg/kg), respectively. The administration site was pressed for 30 s after injection to stop the drug solution diffusing through the blood vessel. On 2, 7, 14, 21 days after injection, one rabbit of each group was sacrificed. The auricular brim veins were treated by Formalin-Fixed and Parrffin-Embedded method and then stained by H&E and watched under a light microscope to assess the effect of chemoembolization.

## Results and discussion

### Formulation building and Box–Behnken design

In this study, 17 experiments were conducted. The selected responses were cumulative release percentage of PYM in 1 day (Y_1_), 9 days (Y_2_) and the rate constant *k* (Y_3_). Higher cumulative release percentage of PYM in 9 days and larger rate constant *k* were better for the preparation controlling the PYM sustainedly release up to 9 days. An initial undesired fast release of the drug from liposomal thermogels may lead to problems of toxicity (Yan et al., 2017). Thus, the adequate control of the release rate in the initial stage is one of the critical problems in the current research. Response data for all experimental runs of Box–Behnken experimental design were shown in Table 3. The values of response Y_1_, Y_2_ and Y_3_ were separately in the range of 15.04 to 42.90, 98.96 to 62.18, and 0.0297 to 0.0823. The ratio of maximum to minimum for both the responses Y_1_, Y_2_ and Y_3_ were 2.85, 1.59 and 2.77, respectively. These results therefore implied that power transformation had low or no effect on the obtained values.

**Table 3.** Observed responses for the Box–Behnken design..

Std/Run	Independent variables				Dependent variables		
	Mode	X_1_	X_2_	X_3_	Y_1_	Y_2_	Y_3_
9/7	0/−1/−1	4	1.8	6	38.26	97.06	0.0564
10/5	0/+1/−1	4	2.2	6	24.31	82.65	0.0458
11/3	0/−1/+1	4	1.8	14	33.45	94.78	0.0711
12/15	0/+1/+1	4	2.2	14	17.89	68.54	0.0688
5/4	−1/0/−1	2	2.0	6	37.12	98.04	0.0419
6/12	+1/0/−1	6	2.0	6	42.90	98.58	0.0520
7/16	−1/0/+1	2	2.0	14	29.78	89.04	0.0393
8/9	+1/0/+1	6	2.0	14	34.67	92.35	0.0816
1/17	−1/−1/0	2	1.8	10	35.24	96.87	0.0459
2/2	+1/−1/0	6	1.8	10	40.03	98.96	0.0774
3/1	−1/+1/0	2	2.2	10	15.04	62.18	0.0297
4/10	+1/+1/0	6	2.2	10	20.56	70.63	0.0806
14/6	0/0/0	4	2.0	10	19.48	85.74	0.0823
17/8	0/0/0	4	2.0	10	18.33	83.66	0.0659
16/11	0/0/0	4	2.0	10	19.09	84.79	0.0741
15/13	0/0/0	4	2.0	10	18.97	85.27	0.0692
13/14	0/0/0	4	2.0	10	19.20	82.23	0.0781

X_1_ is the factor of strength of the preparation, X_2_ is the factor of CS amount, X_3_ is the factor of GP content, Y_1_ is the response of cumulative release percentage of PYM in 1 day, Y_2_ is the response of cumulative release percentage PYM in 9 days, Y_3_ is the response of rate constant *k.*

### Model fitting and analysis

All the response data observed from 17 formulations accorded with first order, two-factor interaction and quadratic models. Model selection for response analysis was carried out based on the sequential model sum of squares, lacking fit test and model summary statistics. The quadratic model was selected for all analyzing responses Y_1_, Y_2_ and Y_3_ for the reason that the Prob > F value of *p* < .0001, low standard deviation, high R-squared value and lower predicted residual error sum of square (PRESS) value. The fit summary for each response was listed in Table 4. A difference less than 0.20 between the ‘Pred R-squared’ value for the responses and the ‘Adj R-squared’ value approved that the model predicted response values well.

**Table 4.** Fit summary for responses Y_1_, Y_2_ and Y_3_.

	Sum of squares			df			F-value			*p* value (Prob > F)					
	Y_1_	Y_2_	Y_3_	Y_1_	Y_2_	Y_3_	Y_1_	Y_2_	Y_3_	Y_1_	Y_2_	Y_3_			
Sequential sum of model square															
Linear	733.52	1498.85	2.88E-03	3	3	3	16.71	10.39	7.27	< .0001	.0009	.0041			
2FI	0.78	48.65	3.71E-04	3	3	3	0.014	0.28	0.92	.9976	.8378	.4669			
Quadratic	174.2	498.87	1.04E-03	3	3	3	26.72	14.94	8.09	.0003	.002	.0112			
Cubic	14.49	69.95	1.27E-04	3	3	3	26.56	11.74	0.97	.0042	.0188	.4894			
Residual	0.73	7.94	1.74E-04	4	4	4	–	–	–	–	–	–			
Total	16435.04	1.39E + 05	0.071	17	17	17	–	–	–	–	–	–			
Lack-of-fit															
Linear	189.47	617.47	1.54E-03	9	9	9	115.78	34.55	3.93	.0002	.0019	.1002			
2FI	188.68	568.82	1.17E-03	6	6	6	172.95	47.75	4.48	< .0001	.0011	.0842			
Quadratic	14.49	69.95	1.27E-04	3	3	3	26.56	11.74	0.97	.0042	.0188	.4894			
Cubic	0	0	0	0	0	0	–	–	–	–	–	–			
Pure Error	0.73	7.94	1.74E-04	4	4	4	–	–	–	–	–	–			
	R-squared			Adjusted R-squared			Predicted R-squared			PRESS			Std. Dev.		
	Y_1_	Y_2_	Y_3_	Y_1_	Y_2_	Y_3_	Y_1_	Y_2_	Y_3_	Y_1_	Y_2_	Y_3_	Y_1_	Y_2_	Y_3_

Model summary statistics															
Linear	0.7941	0.7056	0.6265	0.7466	0.6376	0.5403	0.5772	0.4393	0.395	390.56	1191	2.78E-03	3.82	6.94	0.011
2FI	0.7949	0.7285	0.7072	0.6719	0.5656	0.5314	−0.0195	−0.1638	0.2306	941.73	2472.13	3.54E-03	4.35	7.59	0.012
Quadratic	0.9835	0.9633	0.9345	0.9624	0.9162	0.8502	0.7479	0.4673	0.4991	232.91	1131.57	2.30E-03	1.47	3.34	6.56E-03
Cubic	0.9992	0.9963	0.9621	0.9969	0.985	0.8482	–	–	–	–	–	–	0.43	1.41	6.60E-03

Y_1_ is the response of cumulative release percentage of PYM in 1 day, Y_2_ is the response of cumulative release percentage of PYM in 9 days, Y_3_ is the response of rate constant *k*.

df: degree of freedom; PRESS: predicted residual error sum of squares; statistically significant terms are underlined (*p* value less than .05).

The analysis of variance (ANOVA) was used in determining the significance of the variable effects and their interactions. It validated the model obtained (quadratic model, *p* < .05) for the responses of PYM-loaded liposomal CS/GP thermogels, at the same time, provided key factors affecting these responses. As is observed in Table 5, the CS amount (X_2_) and GP content (X_3_) were considered significant for cumulative release of PYM in 9 days (Y_2_) and rate constant *k* (Y_3_), whereas the PYM concentration (X_1_) and GP content (X_3_) were identified significant for the cumulative release of PYM in 1 day (Y_1_), all of the PYM concentration (X_1_), CS amount (X_2_) and GP content (X_3_) were of significance. Details of ANOVA for response Y_1_, Y_2_ and Y_3_ were presented in Table 5.

**Table 5.** The analysis of variance for responses Y_1_, Y_2_ and Y_3_.

ANOVA	Sum of squares			df			F-value			*p*-value (Prob > F)		
	Y_1_	Y_2_	Y_3_	Y_1_	Y_2_	Y_3_	Y_1_	Y_2_	Y_3_	Y_1_	Y_2_	Y_3_
Model	908.5	2046.38	4.29E-03	9	9	9	46.45	20.43	11.09	< .0001	.0003	.0022
X_1_	50.85	22.41	2.27E-03	1	1	1	23.4	2.01	52.8	.0019	.1988	.0002
X_2_	598.23	1343.43	8.39E-05	1	1	1	275.26	120.74	1.95	<.0001	<.0001	.2054
X_3_	84.44	133.01	5.23E-04	1	1	1	38.85	11.95	12.16	.0004	.0106	.0102
X_1_X_2_	0.13	10.11	9.41E-05	1	1	1	0.061	0.91	2.19	.8116	.3722	.1827
X_1_X_3_	1.60E-03	3.55	2.59E-04	1	1	1	7.36E-04	0.32	6.03	.9791	.5897	.0438
X_2_X_3_	0.65	34.99	1.72E-05	1	1	1	0.3	3.14	0.4	.602	.1195	.547
X_1_^2^	39.7	10.66	5.26E-04	1	1	1	18.27	0.96	12.22	.0037	.3602	.0101
X_2_^2^	80.3	471.89	7.96E-05	1	1	1	36.95	42.41	1.85	.0005	.0003	.216
X_3_^2^	61.78	16.94	3.45E-04	1	1	1	28.43	1.52	8.01	.0011	.257	.0254
Residual	15.21	77.89	3.01E-04	7	7	7	–	–	–	–	–	–
Lack of Fit	14.49	69.95	1.27E-04	3	3	3	26.56	11.74	0.97	.0042	.0188	.4894
Pure Error	0.73	7.94	1.74E-04	4	4	4	–	–	–	–	–	–
Cor Total	923.72	2124.26	4.60E-03	16	16	16	–	–	–	–	–	–

X_1_ is the factor of PYM concentration, X_2_ is the factor of CS amount, X_3_ is the factor of GP content, Y_1_ is the response of cumulative release percentage of PYM in 1 day, Y_2_ is the response of cumulative release percentage of PYM in 9 days, Y_3_ is the response of rate constant *k*.

The resulted equations for responses Y_1_, Y_2_ and Y_3_ were stated below:

$${\text{Y}}_{\text{1}}=\text{29}.0\text{1}+\text{2}.{\text{52X}}_{\text{1}}-\text{8}.{\text{65X}}_{\text{2}}-\text{3}.{\text{25X}}_{\text{3}}+0.{\text{18X}}_{\text{1}}{\text{X}}_{\text{2}}-0.0{\text{2X}}_{\text{1}}{\text{X}}_{\text{3}}-0.{\text{4X}}_{\text{2}}{\text{X}}_{\text{3}}+\text{3}.0{\text{7X}}_{\text{1}}{}^{\text{2}}-\text{4}.{\text{37X}}_{\text{2}}{}^{\text{2}}+\text{3}.{\text{83X}}_{\text{3}}{}^{\text{2}}$$

$${\text{Y}}_{\text{2}}=\text{94}.\text{34}+\text{1}.{\text{67X}}_{\text{1}}-\text{12}.{\text{96X}}_{\text{2}}-\text{4}.0{\text{8X}}_{\text{3}}+\text{1}.{\text{59X}}_{\text{1}}{\text{X}}_{\text{2}}+0.{\text{94X}}_{\text{1}}{\text{X}}_{\text{3}}-\text{2}.{\text{96X}}_{\text{2}}{\text{X}}_{\text{3}}-\text{1}.{\text{59X}}_{\text{1}}{}^{\text{2}}-\text{1}0.{\text{59X}}_{\text{2}}{}^{\text{2}}+\text{2}.0{\text{1X}}_{\text{3}}{}^{\text{2}}$$

$${\text{Y}}_{\text{3}}=0.0\text{74}+0.0{\text{17X}}_{\text{1}}-0.00{\text{3237X}}_{\text{2}}+0.00\text{8}0{\text{88X}}_{\text{3}}+0.00{\text{485X}}_{\text{1}}{\text{X}}_{\text{2}}+0.00\text{8}0{\text{5X}}_{\text{1}}{\text{X}}_{\text{3}}+0.00\text{2}0{\text{75X}}_{\text{2}}{\text{X}}_{\text{3}}-0.0{\text{11X}}_{\text{1}}{}^{\text{2}}-0.00{\text{4347X}}_{\text{2}}{}^{\text{2}}-0.00\text{9}0{\text{48X}}_{\text{3}}{}^{\text{2}}$$

A plus sign represents a synergistic effect, conversely a minus sign shows an antagonistic effect. In case of Y_1_, positive coefficients of X_1_ in the model made reference to an increasing trend of PYM releasing in 1 day when the concentration of PYM increased. Likewise, the negative coefficients of X_2_ and X_3_ showed the decrease of PYM releasing in 1 day with increasing tendency of respective factors. In the matter of Y_2_, the negative coefficients of X_2_ and X_3_ in the model referred to that more PYM released in 9 days when the amounts of CS and GP decreased. Similarly, the positive coefficients of X_1_ suggested the enhanced release of PYM in 9 days with the increasing contents of respective factors. In case of Y_3_, positive coefficients of X_1_ and X_3_ displayed a promoting action of relevant factors on the rate constant k, and the minus sign of X_2_ illustrated that the rate constant k reduced as the amount of CS rose.

A noteworthy interaction effect of X_1_X_3_ could be observed for response Y_3_. This result might be attributed to the electro-static binding between ammonium groups of PYM liposome and phosphate groups of GP. The binding force closely associated with the gelification of the CS/GP *in situ* gel system has been speculated as: (a) the enhanced interchain hydrogen bonds of CS as a result of the decline of electro-static repulsive-force due to the neutralization reaction between GP and CS; (b) the electro-static pull reaction between ammonium groups of CS and phosphate groups of GP, and (c) the hydro-phobic reaction between intermolecular of CS (Fabiano et al., 2017). The competition relationship of PYM liposome/GP electro-static attraction and CS/GP interaction may break the sol-gel change by the above hypothesis (a) and (b), which can lead to a boosted initial fractional release.

### Evaluation of contour plots and response surface

**Figure 1.**
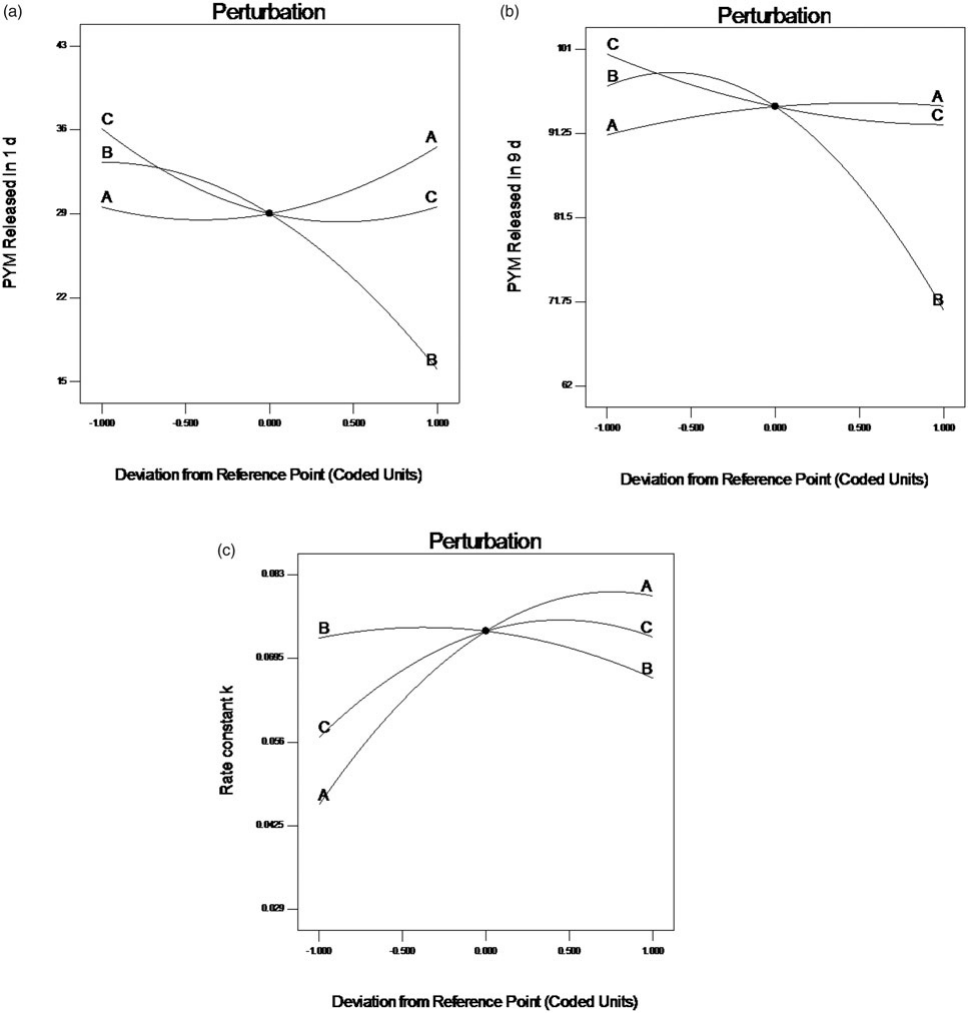
Perturbation plots showed the effects of X_1_ (A), X_2_ (B) and X_3_ (C) on the responses Y_1_ (a), Y_2_ (b) and Y_3_ (c). X_1_ is the factor of drug loading of the preparation, X_2_ is the factor of CS amount, X_3_ is the factor of GP content, Y_1_ is the response of cumulative release percentage of PYM in 1 day, Y_2_ is the response of cumulative release percentage of PYM in 9 days, Y_3_ is the response of rate constant *k*.

Perturbation graphs (Figure 1) were plotted to obtain the most influencing factors to responses. A steep curvature implies that a response is sensitive to a factor, whereas a relatively flat one means insensitive. In case of response Y_1_ (Figure 1(a)), factor B displayed a steep curvature, on the opposite, factor A and C both exhibited a slight slope. The result indicated that factor B was the most influential parameters of response. Whereas in case of response Y_2_ (Figure 1(b)) and Y_3_ (Figure 1(c)), factor B displayed steep curvatures compared with A and C in Figure 1(b), and factor A exhibited steep curvatures comparing to factor B and C in Figure 1(c). These results are consistent with the outcome from the ANOVA.

**Figure 2.**
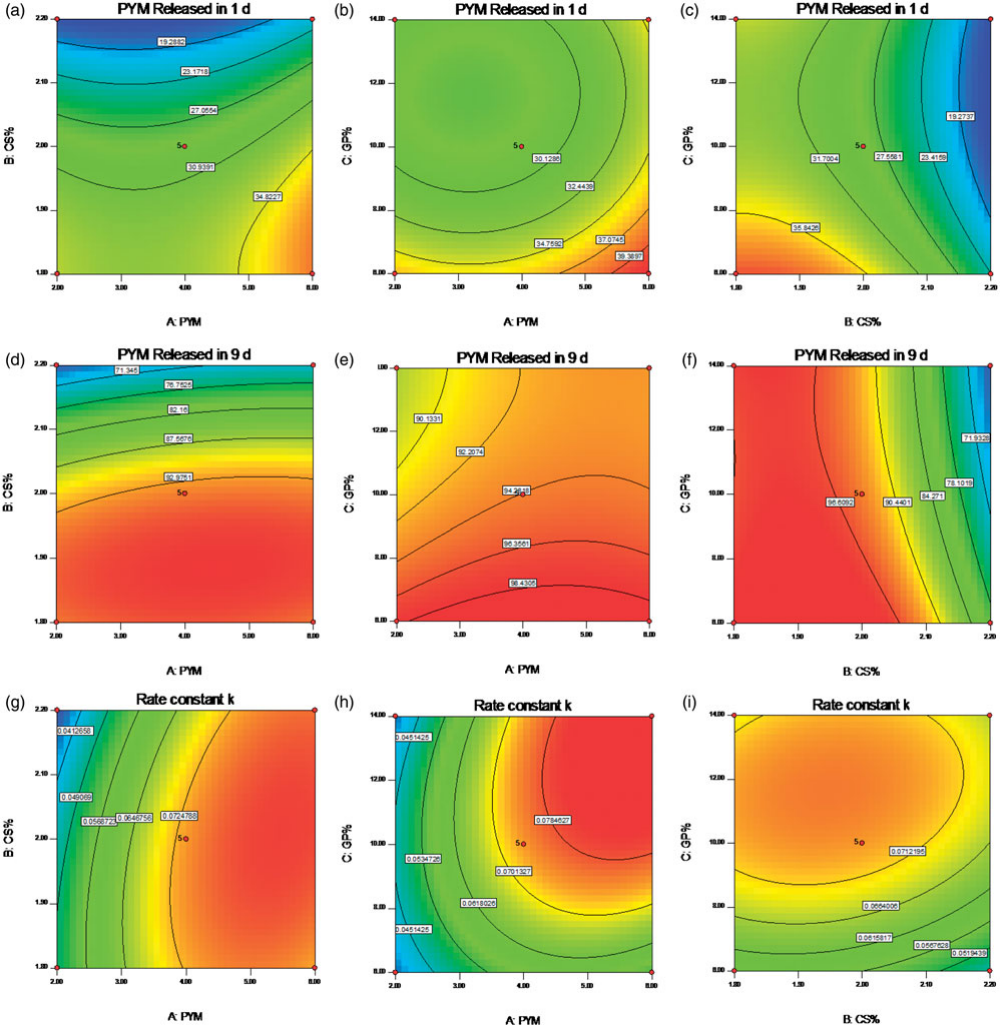
(a–i) Contour plots showed the effects of X_1_(A), X_2_(B) and X_3_(C) on the response Y_1_, Y_2_ and Y_3_. X_1_ is the factor of concentration of PYM, X_2_ is the factor of CS amount, X_3_ is the factor of GP content, Y_1_ is the response of cumulative release percentage of PYM in 1 day, Y_2_ is the response of cumulative release percentage of PYM in 9 days, Y_3_ is the response of rate constant k.

**Figure 3.**
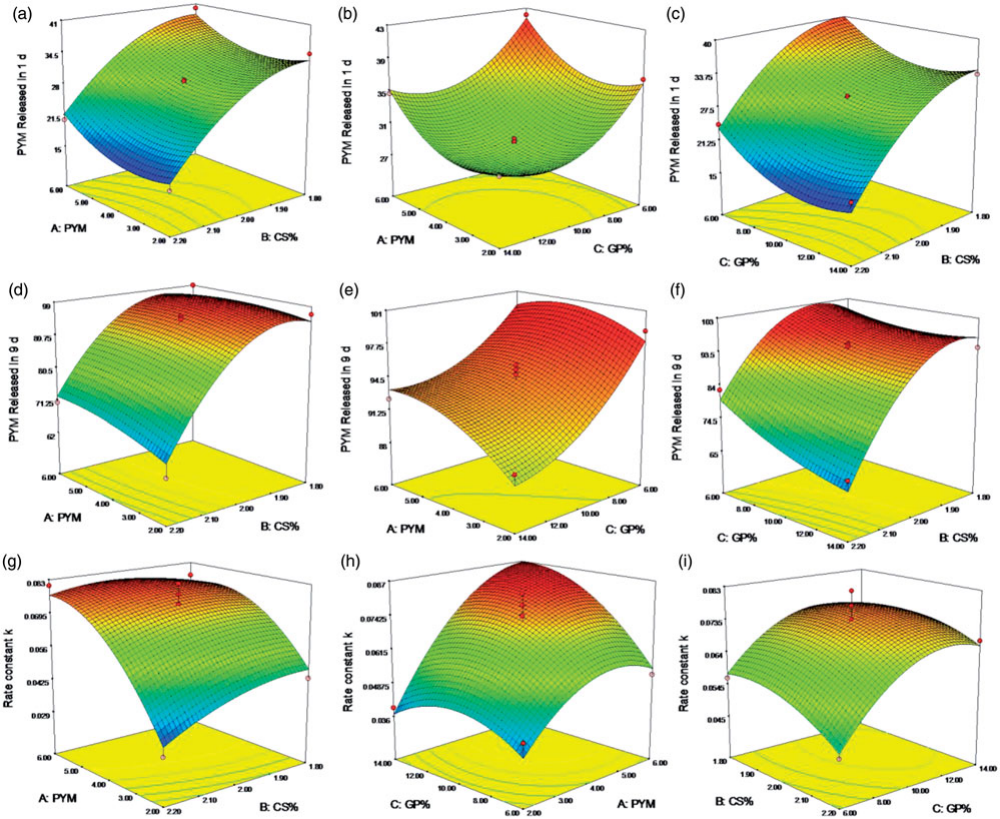
(a–i) Response surface plots showed the effects of X_1_(A), X_2_(B) and X_3_(C) on the response Y_1_, Y_2_ and Y_3_. X_1_ is the factor of concentration of PYM, X_2_ is the factor of CS amount, X_3_ is the factor of GP content, Y_1_ is the response of cumulative release percentage of PYM in 1 day, Y_2_ is the response of cumulative release percentage of PYM in 9 days, Y_3_ is the response of rate constant *k*.

A clear function of factors on responses was revealed in the two-dimension contour plots and three-dimension response surface plots (Figure 2 and Figure 3), which were plotted based on the ANOVA and perturbation plot (Shaikh et al., 2017). In all figures, we kept one factor at zero level. A nonlinear relationships of the all variables in the case of Y_1_, Y_2_ and Y_3_ was indicated in Figure 2, even more distinct in Figure 3. The relative high similarity of contour plots and response surface plots of response Y_2_ and Y_3_ indicated similar release mechanism, both responses were consistent with the prediction of the drug release behavior regardless of the initial release extent. Overall, there was a positive correlation between the increase of GP content and the raise of the initial PYM release and rate constant *k*, which further checked on the guess that the sol-gel change mechanism of the CS/GP *in situ* gel system was nucleation, growth and gelification finish. This reaction process occurred during the formation of microstructure of the CS/GP *in situ* gel with changes of the system temperature. In the nucleation process, the polymer system was constituted by numerous small parts that would grow and aggregate as the extension of reaction time, then leaded to a heterogeneous mixture of the thermogels. It was proved that a minimum concentration of GP was necessary for the gelification of CS/GP thermogels system. And then, electro-static pull reaction between ammonium of CS and phosphate of GP could lead to massive interchain hydrogen-bonding effects (Salis et al., 2015).

### Optimization of PYM liposomal thermogels

**Figure 4.**
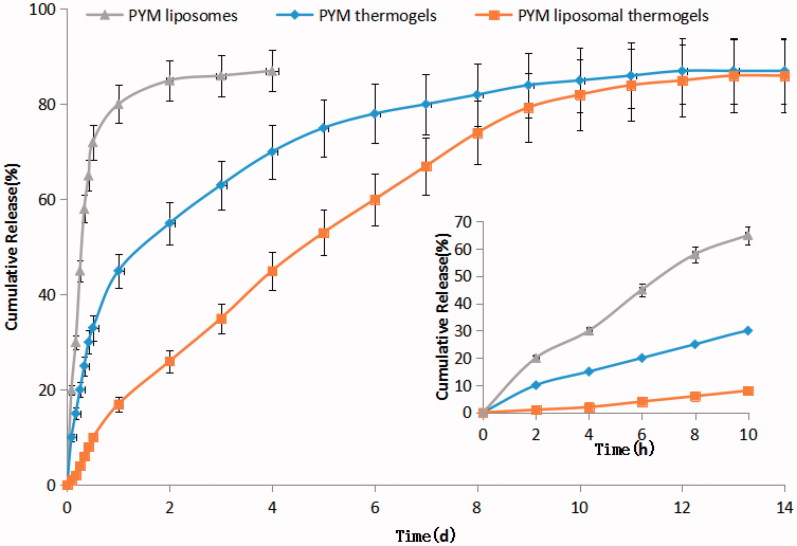
PYM *in vitro* release profile from PYM liposomes, PYM thermogels and PYM liposomal thermogels (*n* = 3).

An optimal formulation was expected to obtain the minimum drug release in 1 day (Y_1_) and maximum drug release in 9 days (Y_2_), at same time maximized rate constant (Y_3_). All the formulation variables were investigated to establish a desirability function, further to forecast a desirable scope of variables which could constitute a optimal formulation. The objective function is as follow:

$$\text{D }=\;{\left({\text{d}}_{\text{1}}{\text{d}}_{\text{2}}{\text{d}}_{\text{3}}\cdot \cdot \cdot {\text{d}}_{\text{n}}\right)}^{\text{1}/\text{n}}$$

where di is the desirable scope for respective response, n is the total number of responses in the experiment (Cong et al., 2017). The desirable ranges for CS amount and GP content are from zero to one, respectively. The optimized ingredients were presented in Table 5. In summary, an optimal formulation of PYM-loaded liposomal CS/GP thermogels for VM should be composed of PYM 4.68 mg/mL, CS 2.05% (w/v), and GP 11.57% (w/v), producing *in situ* gels with 17.24% PYM released in 1 day and 80.77% PYM released in 9 days, and rate constant of 0.0808. The drug release profile of PYM liposomes, PYM thermogels and PYM liposomal thermogels were determined in three parallel tests (Figure 4) for verifying the availability of the computed optimal factors and predicted responses. The result confirmed that the PYM liposomal thermogels was able to release PYM sustainedly up to 14 days without obvious burst release in the initial stage, which was much better than that of PYM liposomes or PYM thermogels. Table 6 revealed that the measured drug release values of the optimized formulation was highly close to the values of the predicted ones with low deviations, suggesting that the optimized formulation is reliable and reasonable.

**Table 6.** The predicted and experimental response values of the optimized formulations.

optimum conditions	Level	Measured responses	Predicted values	Experimental values	Bias (%)
X_1_	4.68	Y_1_	17.2411	16.9741	1.55
X_2_	2.05	Y_2_	80.7707	79.3259	1.79
X_3_	11.57	Y_3_	0.0808058	0.0800025	1.00

X_1_ is the factor of PYM concentration, X_2_ is the factor of CS amount, X_3_ is the factor of GP content, Y_1_ is the response of cumulative percentage PYM released in 1 day, Y_2_ is the response of cumulative percentage PYM released in 9 days, Y_3_ is the response of rate constant *k*.

Bias was calculated as (predicted value − observed value)/predicted value ×100%.

### Morphological changes of EA.hy926 cells

**Figure 5.**
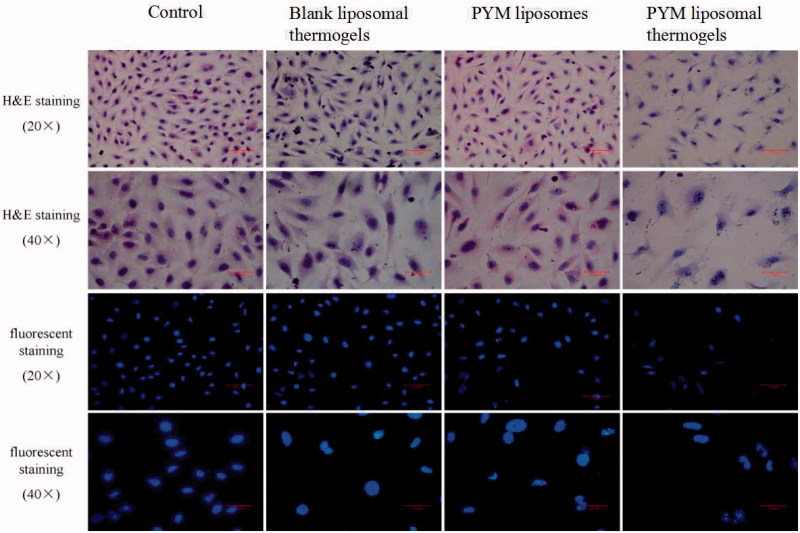
Morphological changes of EA.hy926 cells exposed to blank liposomal thermogels, PYM liposomes and PYM liposomal thermogels for 24 h. Cells were stained by H&E and fluorescent dye, respectively.

Figure 5 exhibited the morphological changes of EA.hy926 cells exposed to PYM liposomes, PYM liposomal thermogels and blank liposomal thermogels. Cells were stained with H&E and fluorescent dye, respectively. The cells in blank liposomal thermogels group grew well with high proliferation rate and arranged closely for 24 h, showing no obvious morphological changes compared with the control group. On the contrary, typical apoptosis morphological features including cell shrinkage and condensation and chromatin margination were widely observed in cells treated with PYM liposomes and PYM liposomal thermogels after 24 h, demonstrating obvious morphological changes compared to the control group.

### Cell cycle analysis and apoptosis assay

**Figure 6.**
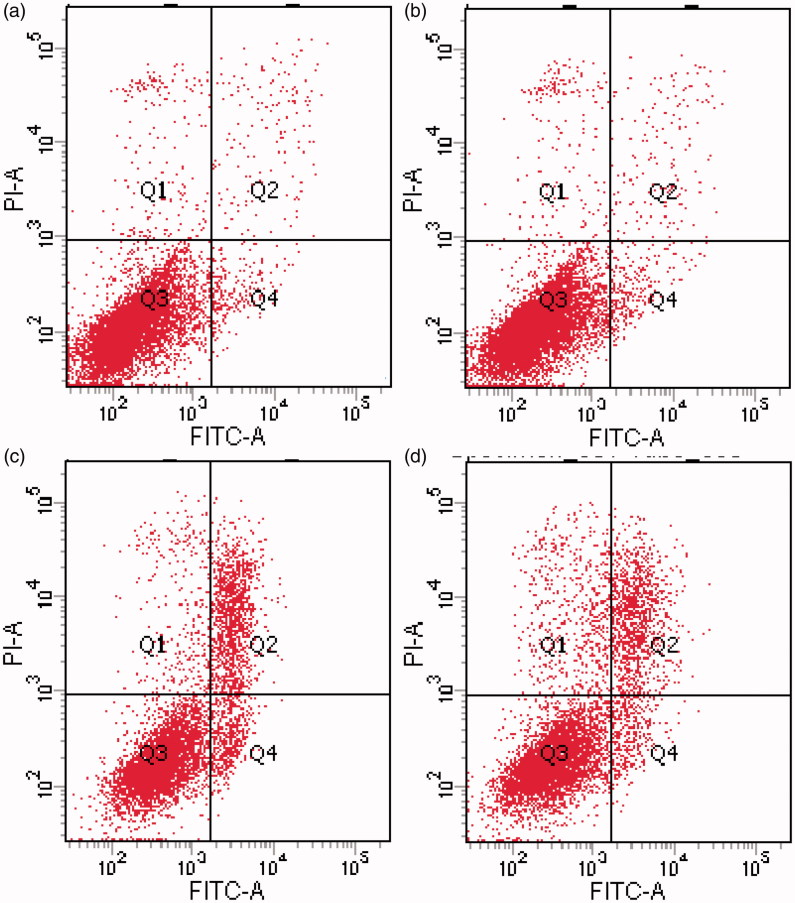
Effects of PYM on EA. hy926 *in vitro* treated with (a) control, (b) blank liposomal thermogels, (c) PYM liposomes(1 μg/mL), (d) PYM liposomal thermogels (1 μg/mL) for 24 h, discriminated by FITC-annexin V & PI.

To further access the cytotoxicity of ingredients of liposomal thermogels, cell cycle analysis and apoptosis assay of EA.hy926 cells incubated with different preparations were carried out. As shown in Table 7 and Figure 6, blank liposomal thermogels group exhibited no differences with control in cell cycle and apoptosis assay, consistent with the result of morphological changes, which suggested that the ingredients selected for liposomal thermogels was cell-compatible and the cytotoxicity was caused by PYM and not by liposomal thermogels formulation components. Similarly, cell division G0/G1 stage proportion of PYM liposomes and PYM liposomal thermogels group slightly reduced, showing no significant statistical differences with the control group (*p* > .05). However, there was an obvious decrease in the proportion of cell division S phase and a marked increase in the percentage of G2/M phase for PYM liposomes and PYM liposomal thermogels group, with significant statistical differences (*p* < .05). The above results confirmed that PYM liposomes and PYM liposomal thermogels effectively inhibit the growth of EA.Hy926 by blocking the cell division in G2/M phase. As displayed in Figure 6 and Table 8, PYM liposomes and PYM liposomal thermogels had significant induction effect of apoptosis and necrosis, which obviously enhanced with the increase of PYM concentration and action time.

**Table 7.** Effects of PYM liposomes, PYM liposomal thermogels and blank liposomal thermogels on the cell cycle of EA.hy926 (*n* = 3,10 μg/mL,24 h).

	G0/G1(%)	S(%)	G2/M(%)
Control	44.8 ± 0.7	43.5 ± 1.6	11.7 ± 0.6
Blank liposomal thermogels	46.5 ± 1.2	40.0 ± 1.0	13.5 ± 0.4
PYM liposomes	41.9 ± 0.6	17.1 ± 0.3	41.0 ± 1.1
PYM liposomal thermogels	42.3 ± 1.4	19.8 ± 0.6	37.9 ± 0.9

**Table 8.** Apoptosis rate & Necrosis rate of EA.hy926 *in vitro* treated with blank liposomal thermogels, PYM liposomes and PYM liposomal thermogels (*n* = 3, 1 μg/mL & 10 μg/mL, 24 h & 48 h).

	24 h		48 h	
	Apoptosis (%)	Necrosis (%)	Apoptosis (%)	Necrosis (%)
Control	4.3 ± 0.2	2.2 ± 0.3	4.6 ± 0.3	2.5 ± 0.5
blank liposomal thermogels	4.5 ± 0.4	2.1 ± 0.5	4.5 ± 0.1	2.2 ± 0.4
PYM liposomes (1 μg/mL)	26.2 ± 1.0	4.7 ± 0.8	29.4 ± 1.3	13.6 ± 0.7
PYM liposomes (10 μg/mL)	30.8 ± 1.3	12.2 ± 0.9	32.3 ± 1.6	20.5 ± 0.7
PYM liposomal thermogels (1 μg/mL)	21.3 ± 0.6	8.4 ± 1.0	27.6 ± 1.4	15.4 ± 1.1
PYM liposomal thermogels (10 μg/mL)	29.5 ± 1.4	17.8 ± 1.2	33.9 ± 1.1	22.1 ± 1.2

### Pharmacokinetics in rabbits

**Figure 7.**
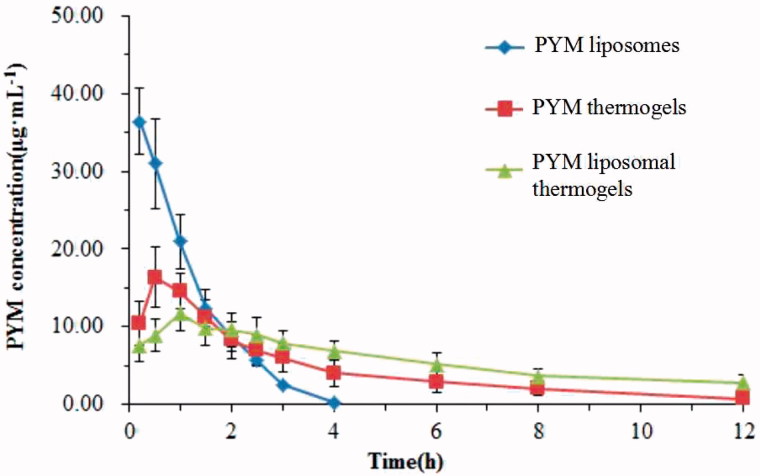
Mean blood concentration–time curve of PYM in rabbits after administration of PYM liposomes, PYM thermogels and PYM liposomal thermogels (Each value represents the mean ± SD, *n* = 6).

Plasma concentration-time profiles of PYM after injection of PYM liposomes, PYM thermogels and PYM lipsomal thermogels (Figure 7) clearly demonstrated that PYM liposomal thermogels had significant sustained-release characteristics compared with PYM thermogels and PYM liposomes. The main pharmacokinetic parameters of three groups are shown in Table 9. The half-time (t_1/2_) of PYM liposomal thermogels was almost 2.4 times and 9.3 times longer than that of PYM thermogels and PYM liposomes, respectively. MRT of PYM liposomal thermogels was significantly prolonged (8.90 ± 2.25 h) compared to that of PYM thermogels (3.61 ± 0.34 h) and PYM lipsomes(1.08 ± 0.09 h). In addition, the decrease of *C*_max_ (from 37.08 ± 4.60 to 10.93 ± 2.19 μg·mL^−1^) and the delay in *t*_max_ (from 0.20 to 1.00 h) also indicated the liposomal thermogels had a better sustained delivery of PYM.

**Table 9.** Pharmacokinetic parameters of PYM liposomes, PYM thermogels and PYM liposomal thermogels.

Parameter	Units	PYM liposomal thermogels	PYM thermogels	PYM liposomes
*C*_max_	μg·mL^−1^	10.93 ± 2.19	16.81 ± 2.95	37.08 ± 4.60
*t*_1/2_	h	6.54 ± 1.75	2.69 ± 0.23	0.70 ± 0.11
AUC_0–t_	μg·L^−1^·h	70.44 ± 15.79	50.93 ± 11.82	51.60 ± 7.30
AUC_0–∞_	μg·L^−1^·h	91.36 ± 22.87	56.29 ± 17.24	63.22 ± 9.16
MRT_0–∞_	h	8.90 ± 2.25	3.61 ± 0.34	1.08 ± 0.09

### Chemoembolization studies in rabbits

**Figure 8.**
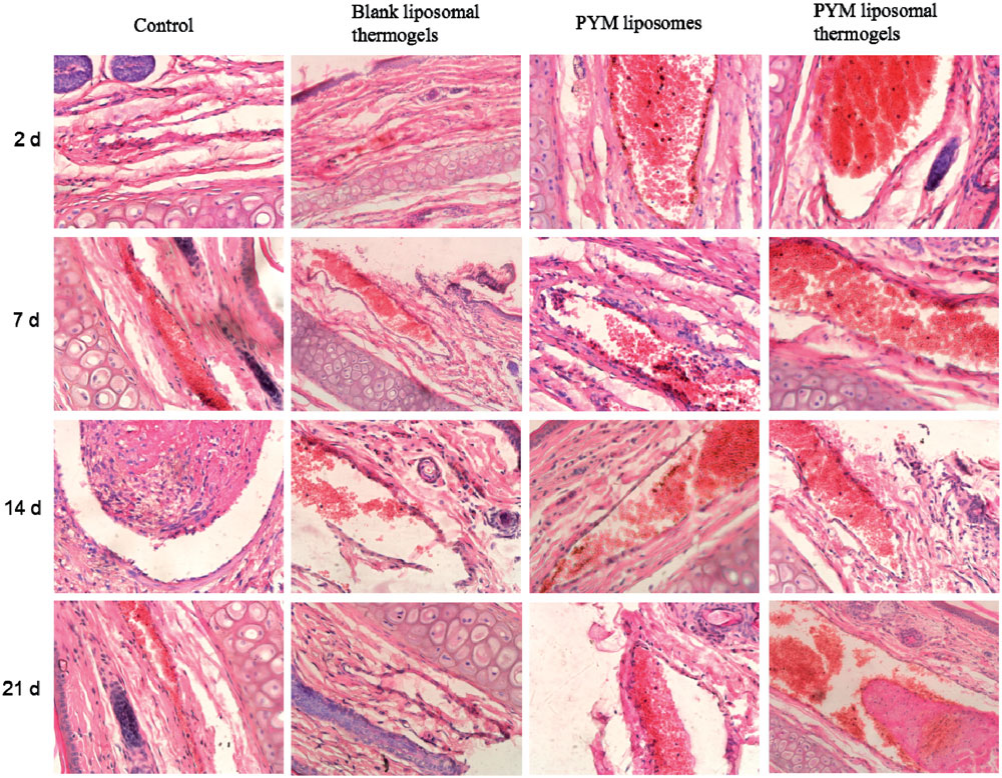
Histological sections of rabbit ear veins after administration of normal saline solutions, blank liposomal thermogels, PYM liposomes and PYM liposomal thermogels for 2, 7, 14, 21 days (40×).

Figure 8 showed photomicrograph of crosscut tissues of rabbit’s ear side veins stained by H&E after injection of normal saline solutions, blank liposomal thermogels, PYM liposomes and PYM liposomal thermogels. In the blank liposomal thermogels group, mild edema appeared after administration for 2 days. Whereas no obvious histological change was found after injection for 7 days, which confirmed that blank liposomal thermogels had considerable bio-compatibility for application in the injectable controlled drug release preparation. The result after treatment of PYM liposomes showed that vessel endothelial cells were swelling and diapedesis appeared obviously for the beginning 2 days. While, no other histological change of PYM liposomes group was discovered on the next 7–21 days, demonstrating that PYM liposomes had spread with blood flowing and local PYM concentration in the administration site decreased rapidly. In the PYM liposomal thermogels group, vessel embolism caused by PYM liposomal thermogels could be found obviously afer injection for the beginning 2 days. Vessel endothelial cells were exfoliated on the 7th day. After two weeks, the venous wall was thickened and the vessel lumen was narrowed. Obvious venous occlusion were discovered on the 21st day.

It could be concluded that, compared with PYM liposomes, PYM liposomal thermogels displayed much better effect for therapy of VM. PYM-loaded liposomal thermogels showed multiple actions during the process of embolization after administration into auricular brim veins. At the beginning, vessel occlusion happened rapidly after the sol–gel change of the liposomal thermogels. And then, the hyperplasia of vessel endothelial cells formed along with the controlled and localized release of PYM from liposomal thermogels. The liposomal thermogels were degraded gradually by the lysozyme *in vivo* and then PYM was released sustainedly, which could make a local high level of PYM and increase the MRT of PYM and finally improve the curative effect.

## Conclusions

In the current study, an *in vitro* and *in vivo* evaluation of PYM-loaded liposomal CS/GP *in situ* thermogels was conducted for the treatment of VM. The liposomal CS/GP *in situ* thermogels for the controlled delivery of PYM were prepared and optimized by Box–Behnken experimental design. The optimal formulation was composed of PYM 4.68 mg/mL, CS 2.05% (w/v), and GP 11.57% (w/v), producing *in situ* gels with 17.24% and 80.77% PYM released in 1 day and 9 days, respectively, and rate constant of 0.0808. The induction effects of apoptosis and necrosis were both observed for PYM-loaded liposomal CS/GP *in situ* thermogels in inhibiting the growth of EA. hy926 cells. The rabbit *in vivo* pharmacokinetics and *in vivo* embolization study of PYM liposomal thermogels, compared with PYM thermogels and PYM liposomes, indicated that PYM liposomal thermogels could make sustained and localized drug release for a longer time, which reached the purpose of controlled drug delivery. Hence, it is believed that this study can facilitate the exploration of the interaction between drug and liposomal thermogels components, to offer an optimized PYM-loaded liposomal CS/GP thermogels for interventional embolization therapy of VM.
